# Transmission Raman Spectroscopy for Inner Layers Chemical Analysis of Fresh Produce

**DOI:** 10.3390/s25092805

**Published:** 2025-04-29

**Authors:** Rani Arielly

**Affiliations:** Institute of Agricultural and Biosystems Engineering, Agricultural Research Organization–Volcani Institute, Rishon LeZion 7505101, Israel; rania@volcani.agri.gov.il

**Keywords:** postharvest, food quality, food safety, optics, Raman spectroscopy, SERDS

## Abstract

**Highlights:**

**What are the main findings?**

- Optimal design aspects and optical parameters for Inner Layers Chemical Analysis of Fresh Produce with transmission Raman spectroscopy

**What is the implication of the main finding?**

- Non-destructive chemical analysis of fresh produce inner layers;
- Technology for detecting internal properties and problems in agricultural produce.

**Abstract:**

Identification of chemical properties in the inner tissues of fresh produce would enable us to identify major issues plaguing the agriculture supply chain, like off-flavors and core rot since these are caused by or accompanied by known chemical elements. We show the development of transmission Raman spectroscopy system for identifying these elements by addressing several issues: we located an optimal spectral window by conducting optical attenuation measurements and calculated the required LASER power in that range. For apple tissues, this optical window was found in the 700–950 nm range, and the required LASER power range was calculated to be in the 40–700 mW range. We also calculated that the optimal shifted-excitation Raman difference spectroscopy wavelengths should be separated by 0.7 nm in order to optimally produce narrow and high-intensity Raman peak features and eliminate the competing fluorescence signal. Finally, we provide a complete optical system design with exact optimal parameters. In contrast to other fields like pharmaceuticals and medicine, transmission Raman spectroscopy has not been applied extensively in agriculture. Therefore, this study fills a gap in that field’s applicability.

## 1. Introduction

**Figure 1:**
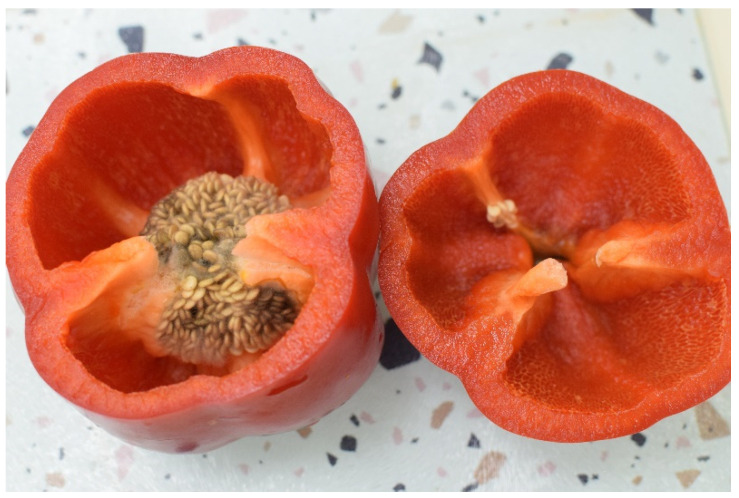
Placenta rot in bell peppers caused by the phytopathogenic fungi Alternaria alternate. Photo credit for B, F. Cohen and C. Ziv.

Pathogenic damage to fresh produce that cannot be diagnosed externally is an unsolved problem for reducing food waste and economic damage [1,2,3,4,5]. Postharvest decay is caused by dormant pathogens, meaning that it is a condition in which the produce is infected within the orchard. The infection is not expressed during the growth period, nor has it been diagnosed at the harvest stage. The development of the disease until signs appear occur during storage. Some pathogens, especially Alternaria and Fusarium in apples and peppers, will infect the produce through the flower cup or leaf column in the fruit, allowing them access to the core of the fruit and the placenta (Figure 1). This core rot is a significant cause of damage to the produce in the global apple and pepper markets, and controlling the infections is not simple because these can occur from the flowering stage and during the development of the fruit [3,4]. The main difficulty in eradicating these diseases is the fact that from the moment of the spores’ penetration into the fruit, they are protected from pesticides applied on the surface, and these are systemic substances that are problematic in use due to their persistence in the fruit and their toxicity to consumers [6]. This becomes even more problematic when exporting produce in large volumes, which require sea freight over many weeks, due to the fact that this long period can allow the infection to spread to the entire container even when refrigerating since some pathogens can thrive at these temperatures. Beyond the economic harm, this also poses a health risk due to the ability of these fungi to produce toxins—mycotoxins, which diffuse through the fruit tissue and contaminate “healthy” parts that look edible [7]. These and other chemical changes to the fruit tissue are associated with the metabolism of the pathogens [8,9,10,11].

For most produce types, the tissue’s molecular content is mostly composed of water (between 78 and 95%) and saccharides such as starch, cellulose, and sugars (between 6 and 21%). The rest of the content will include substances that can be indicative of positive aspects of the produce, such as vitamins, or negative ones, such as ethyl acetate or acetic acid, that the consumer will experience as off-flavor [12,13,14,15]. Identification and quantification of the molecular content can enable the proper marketing of the produce or its elimination when needed.

Identification of chemical compounds or any change in these will usually be performed by measuring their vibrational energies or molecular mass, the latter of which is usually destructive [16,17] but can be very effective and provide us with a reference golden standard. Multiple examples of such studies can be found detailing efficient extraction protocols applied to various fruits and vegetables for detecting fungicides, pesticides, and flavor components [18,19,20,21,22]. If these chemical changes are manifested on the surface of the produce, then current analytical devices can be used to analyze them. If, however, these changes are at the depth of a produce with a small surface-area-to-volume ratio, they cannot reliably be measured with surface sensing methods, such as spectroscopic measurements in the infrared, due to the strong optical attenuation of the tissue, which is dependent on the above-mentioned molecular content [23]. Absorption measurements in the near-infrared range that have become more common recently will often allow deeper penetration of several millimeters below the surface [24], but their analysis will be complicated due to the broad spectroscopic bands arising from harmonics and combination bands of the different modes [25] and the low spectroscopic resolution of the spectrometers. For example, even in the simplest case of the OH stretch band, for the most common SWIR detector that covers the range of 0.85–1.65 μm, a width of $\sim 600\;cm^{-1}$ in the mid-IR range will result in a width of $\sim 1800\;cm^{-1}$ in the NIR range. In fresh produce, due to the high water content, this will be the most intense feature and will cover ~1/3 of the detector’s range. Other water and non-water combinations and harmonics are also possible, which will produce a very smooth and broad spectral shape, dominated by water. Due to this reason, the success of the SWIR analysis will be dependent on the specific goal and sample conditions and processing steps [26,27]. There are several examples of spectroscopic studies that were performed at shorter wavelengths by relying on second-order Raman scattering, but these were also performed by collecting reflected light from the surface, with no attempt to choose a wavelength that best penetrates the tissue [28,29,30]. In the absence of information originating directly from the depth of the produce, current diagnostic attempts rely on information from broad spectral features and spatial information from the tissues adjacent to the surface of the produce and conclude, based on correlations with other destructive measurements, the state at the depth of the produce, and therefore are of low reliability during application [31,32,33,34]. In order to generate data from deep inside the produce, we need a sensor that does not interact strongly with the tissue, and in the case of electromagnetic radiation, a range of wavelengths in which the optical attenuation is weak, i.e., an optical window. Within this optical window we would like to measure spectra resulting from fundamental transitions like in the mid-IR regime. This is achievable via a second-order interaction, which is the basis for Raman spectroscopy [35,36]. This method can provide us with high-quality spectral data from deep inside the produce, but the success of the method may also be hindered by the success in finding an optical window that is transparent enough to compensate for the much lower cross-section of the Raman process compared to first-order processes such as absorption. This measurement method is not utilized but in few fields, such as pharmaceuticals for the analysis of small tablets [37,38,39] and in medicine for identifying breast cancer [40]. The dimensions of the most prevalent tested samples from these examples are very small and their molecular content is made of very few components, which allows the choice from a wide variety of Raman excitation wavelengths. This choice of spectral range becomes more difficult when dealing with agricultural produce sized several cm, which is still unique for this measurement method. Therefore, this method cannot be replicated between fields due to the very different spectral attenuation of the media, and therefore should be adjusted for its use in agriculture. In the following section we will provide details on the required relevant specifications with measurements and calculations to support it.

## 2. Methods and Results

We wish to perform a Raman spectroscopy experiment on the inner layers of fruits. Therefore, in order to be able to irradiate these layers and sense the resulting scattered light, we need this light to be in the wavelength range that the fruits are most transparent.

The attenuation of radiation at some wavelength (λ) through a medium can be described in a differential equation that is solved by this exponential expression.

(1) $$I_{\lambda }=I_{0}\exp\left(-\int \mu_{\lambda }\left(z\right)dz\right)$$

where $I_{0}$ is the initial radiation intensity, $I_{\lambda }$ is the radiation intensity after passing through the medium, $\mu_{\lambda }$ is the attenuation coefficient, and z is the spatial coordinate of the optical path. Many measurement instruments will report the absorbance of the sample ($=\frac{1}{\ln\left(10\right)}\int \mu_{\lambda }\left(z\right)dz$) by knowing the initial and final intensities, and thus, for a uniform sample, it is possible to calculate the attenuation by dividing by the sample’s length.

Although it is possible to find optical attenuation values of agricultural produce and their known components in the literature [13,14,41,42,43,44,45], they are usually limited in the spectral range and do not always calibrate well with each other. In order to locate the ideal spectral window for this application in the broadest range possible (from the UV to the IR), controlled spectrally broad measurements were required.

**Figure 2:**
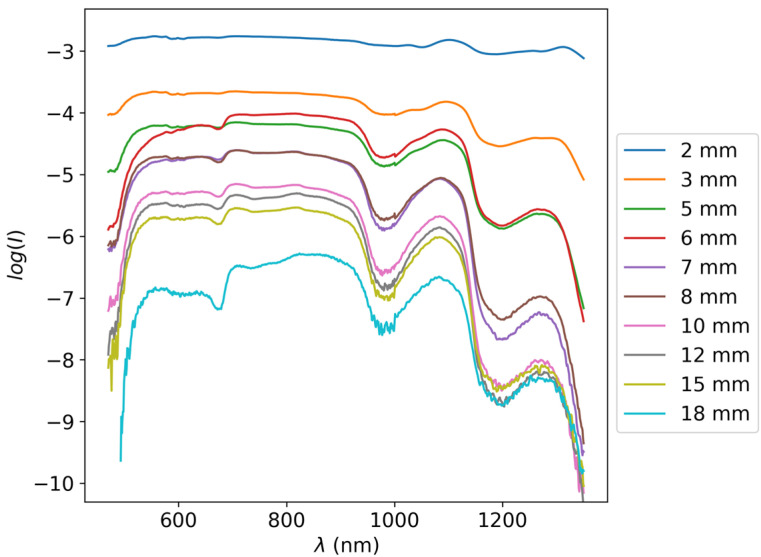
$\log\left(I\right)$ spectra of apple slices of varying thicknesses in the visible and near-infrared spectral regimes. Spectral curves with lower values correspond to thicker slices. The slice thicknesses for all spectra are noted in the legend on the right.

Several apples were cut into slices of varying thicknesses between 0.5 and 20 mm using a slicing machine (Graef Slicer Classic C20; Gebr. Graef GmbH & Co. KG, Arnsberg, Germany) with a slicing resolution of 0.5 mm. The optical attenuation through these slices was measured with Shimadzu UV-1800 UV/Visible Scanning Spectrophotometer (Shimadzu corporation Tokyo, Japan) and again with an Ocean optics LS-1 Tungsten Halogen Light Source (Ocean Optics, Orlando, FL, USA) and ASD FieldSpec 4 spectrometer (Malvern Panalytical, Longmont, CO, USA) [46]. Both instruments were used in order to assess the optical attenuation in the broadest spectral range possible due to several factors that can affect the quality of transmission Raman spectroscopy measurement, from the optical attenuation itself to the wavelength dependency of the Raman cross-section. However, due to the very high attenuation in the UV and near UV optical range, only the ASD FieldSpec 4 measurements were eventually used. An example of the transmittance through apple slices of varying thicknesses can be seen in Figure 2.

**Figure 3:**
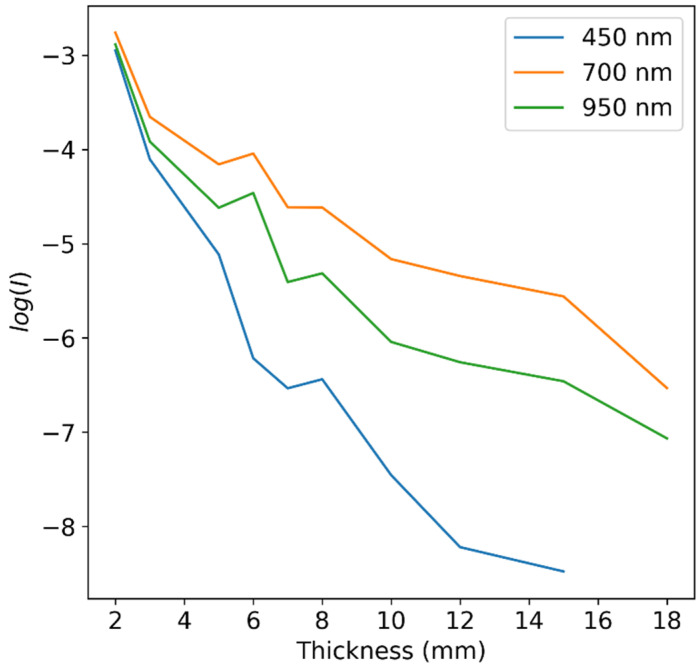
$\log\left(I\right)$ values of apple slices of varying thicknesses at several wavelengths. A general linear trend is evident, with some deviation at higher thicknesses, depending on the wavelength.

By using a log function on Equation (1), we can obtain the absorbance of our samples. For uniform medium, this absorbance is linearly dependent on the thickness of the sample up to certain thicknesses, depending on the wavelength. This relation is experimentally demonstrated on the apple samples data in Figure 3.

**Figure 4:**
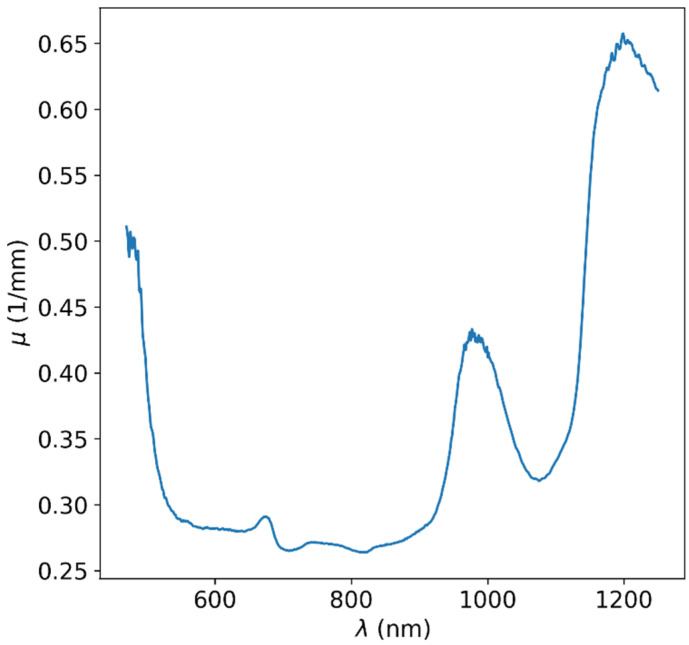
Optical attenuation data for apple. The attenuation increases at wavelengths higher than ~950 nm due to the attenuation of the water component. The attenuation also increases at wavelengths lower than ~550 nm due to the other organic substances of the produce. It is clear that the best spectral window is approximately between 550 and 950 nm.

For each wavelength, the thickness-dependent linear regime was located by calculating $R^{2}$ scores while filtering the higher-thicknesses data points. Values of light attenuation in the material were acquired from the slope of the linear model regression on the automatically filtered data points for each wavelength. The resulting attenuation values as a function of wavelength are shown in Figure 4. Although the total wavelength range of our measurement devices culminated in a 190–2500 nm range, due to high attenuation at some wavelengths, the maximal usable range was only ~400–1250 nm. It is evident that in this usable range, there is a subrange in which the attenuation is minimal and that the attenuation rises sharply at wavelengths higher than ~950 nm due to the higher attenuation of water in this range [23] and at wavelengths shorter than ~550 nm. Thus, it is clear that the best spectral window is approximately between the 550 and 950 nm range, often called the biological window or diagnostic window in medical applications.

**Figure 5:**
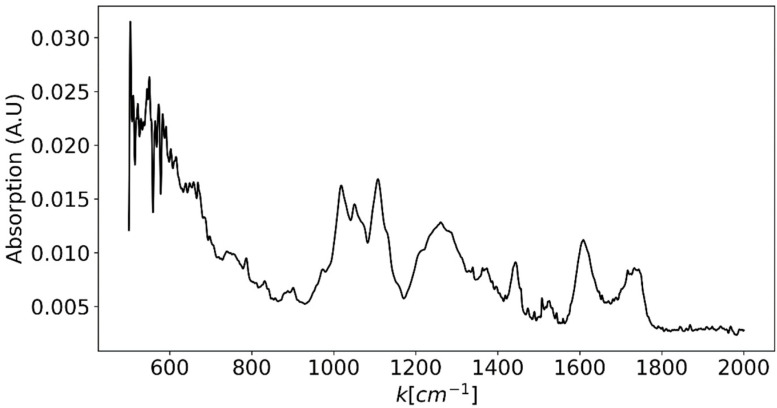
FTIR spectrum of a grape slice after subtraction of the water spectral component. This spectrum is used as a best-case scenario for evaluating the typical line width of spectral features in agricultural produce samples. This line width is estimated as $\sim 30\;cm^{-1}$.

In this spectral range, we should locate a subrange that includes the LASER incident radiation and preferably the entire wavelength range of the scattered light due to vibration excitations up to $k\sim 3750\;cm^{-1}$, which is the end of the H-O stretch mode [47], the highest energy pure common mode. The resulting wavelength (in nm), λ, can be calculated with the following:

(2) $$\lambda =\frac{10^{7}}{\left(\frac{10^{7}}{\lambda_{laser}}-k\right)}$$

where $\lambda_{laser}$ is the LASER’s wavelength. This results in a maximum excitation wavelength of ~700 nm. If, however, one would be satisfied with waiving the functional groups region and only measure the fingerprint region (up to $\sim 1600\;cm^{-1}$), it would be possible to excite up to a wavelength of ~825 nm. However, some important bands that would be missing, for example, in this latter case are the C=C and C=O bands in lipids and the Amide I peak in proteins. In order to properly design the Raman spectroscopic system, we should also estimate the required spectroscopic resolution. Therefore, as a best-case scenario compared to available grating-based spectroscopic equipment, FTIR spectroscopy measurements were performed on several types of produce using ThermoFisher Scientific Nicolet iS50R FTIR Spectrometer (Thermo Fisher Scientific Inc., Waltham, MA, USA) from which the spectroscopic line width was estimated [46]. Since the measurements were dominated by the sample’s water content, in order to better view the more unique features of each sample, the mean spectrum was subtracted from each measurement. FTIR measurement of sliced grape is presented in Figure 5. From these measurements, a line width of $\sim 30\;cm^{-1}$ was estimated. Therefore, in order to fully resolve a single peak’s shape, 10 measurement points were decided upon for each peak, which translates to a spectroscopic resolution of 3 cm^−1^.

Using the above possible wavelength range and required resolution, several wavelength ranges from 600 nm to 950 nm, which should be suitable for a variety of water-based agricultural samples, were calculated along with the required wavelength dispersion for reaching the desired $3\;cm^{-1}$ resolution, and are presented in Table 1. The dispersion is calculated by adding the resolution requirement to the excitation wavenumber and subtracting the resulting wavelength from the original. The number of required pixels is calculated by uniformly dividing the dispersion value across the entire wavelength range.

Designing a spectroscopic system with dispersion values and wavelength ranges, as shown in Table 1, that would be able to measure the desired spectrum in a single shot is possible by combining the right parameter set of focal length (f), grating density (ρ), CCD size (d), and pixel count (N). Several of such combinations are shown in Table 2. These options are based on the most common focal lengths and grating densities and were selected in order to maximize the grating efficiency (based on available gratings specifications [48]). We have used here a CCD with 2048 pixels spread upon a detector size of 27.648 mm. From these options, we can see that in order to capture the full desired spectral range while maximizing efficiency, we should choose a spectrometer with a 300 mm focal length and 300 g/mm grating density. For just measuring the fingerprint region (sometimes at a much better resolution), the table lists other possible configurations, but if one wishes to keep both options, it is clear that a spectrometer with a 300 mm focal length is the best option, coupled with two gratings of 300 and 600 g/mm.

After setting these spectroscopic parameters according to our desired resolution and range, in order to acquire a sufficient signal for the Raman spectrum, we should calculate the total efficiency due to the optical elements, such as the mirrors and grating, and also due to the CCD camera, so we could compensate this if needed by increasing the input power. The quantum efficiency (QE) curve of scientific Si-based CCDs usually descends from its maximal value of above 90% in the visible spectrum to lower values at longer wavelengths. Taking the worst case at our maximum desired wavelength of 950 nm, the QE has a value of ~30%. Using a spectrometer with ρ=300 grooves/mm and 1 μm blaze, we can expect at the desired spectral region a minimum efficiency of ~60% [48]. The mirrors’ efficiency is usually close to 100%, so it will not be taken into consideration. Thus, if no other optical element is present, the total minimal efficiency (χ) of the spectrometer will be ~18%. Another hindrance we should consider from the CCD is its dark current ($I_{dark}$), which has a typical value of 0.002 electron/pixel/sec [49,50]. These parameters will come into play later when evaluating the required LASER power.

In order to estimate the required LASER power for measuring the Raman signal with a decent signal-to-noise ratio (SNR), we will start with a 1D calculation of the total power of the scattered radiation. Let us consider a density function that has a finite value ($\rho_{0}$) within a length, L, and is zero elsewhere.

(3) $$\rho \left(z\right)=\left\{\begin{array}{cc} 0 & z<0\\ \rho_{0} & 0\leq z\leq L\\ 0 & z>L \end{array}\right.$$

Radiation incident upon this density will decrease in intensity from its initial value of $I_{0}$ when progressing through the matter due to different scattering processes represented via the attenuation coefficient μ.

(4) $$I\left(z\right)=\left\{\begin{array}{cc} I_{0} & z\leq 0\\ I_{0}\exp\left(-\mu z\right) & 0<z<L\\ I_{0}\exp\left(-\mu L\right) & z\geq L \end{array}\right.$$

where $I_{0}=\frac{W_{0}}{\pi r_{opt}^{2}}$, $W_{0}$ is the LASER power incident on the sample, and $r_{opt}$ is its beam width. Radiation resulting from Raman scattering will also be scattered until it exits the finite density area. Thus, the total power of the Raman signal observable at z>L in this 1D case will be as follows:

(5) $$P_{1D}=\int_{-\infty }^{\infty }I\left(z\right)\rho \left(z\right)\sigma_{raman}\exp\left(-\mu \left(L-z\right)\right)\;dz=I_{0}\rho_{0}\sigma_{raman}L\exp\left(-\mu L\right)$$

where $\sigma_{raman}$ is the Raman scattering process cross-section. We can expand this to the total 3D case by considering a radial dependence for L, which will affect I and ρ.

(6) $$P=\int dV\;I\left(z,r\right)\rho \left(z,r\right)\sigma_{raman}\exp\left(-\mu \left(L(r)-z\right)\right)\;=2\pi I_{0}\rho_{0}\sigma_{raman}\int_{0}^{r_{opt}}L(r)\exp\left(-\mu L(r)\right)rdr$$

This is performed under the assumption that nonlinear optical propagation paths in the media are insignificant compared to the linear paths due to the strong attenuation.

Assuming spherical symmetry for the fruit, we can express L(r) by using the law of sines:

(7) $$L(r)=2r_{0}\cos\left(\sin^{-1}\left(\frac{r}{r_{0}}\right)\right)$$

where $r_{0}$ is the radius of the finite density region (the fruit), $\rho_{0}$.

If the probability of the radiation of passing the peel is $Q_{peel}$, then assuming that the peel is thin relative to the entire fruit, the output radiation power is $P^{\prime }=PQ_{peel}^{2}$. Values of $Q_{peel}$ were measured alongside the apple slices in the measurement set that is presented in Figure 2. The above statements and equations determine the Raman signal power for a LASER beam incident upon some fruit. We are faced with the potential problem of insufficient Raman signals when the attenuation/dark current is too high or the efficiency/LASER power is too low. Therefore, we wish to write a condition by which to decide the required LASER power, given other set conditions. If we decide that in order to have good results, we should have a minimal threshold for the SNR of 5 (on the time scale of the referenced dark current = 1 s [49]), then we can write the condition

(8) $$P^{\prime }>\frac{5I_{dark}hc}{\chi \lambda }$$

where h is the plank constant, c is the speed of light, and λ is the wavelength. Therefore,

(9) $$W_{0}>\frac{hc}{\lambda }\frac{5I_{dark}r_{opt}^{2}}{\chi Q_{peel}^{2}2\rho_{0}\sigma_{raman}\int_{0}^{r_{opt}}L(r)\exp\left(-\mu L(r)\right)rdr}$$

**Figure 6:**
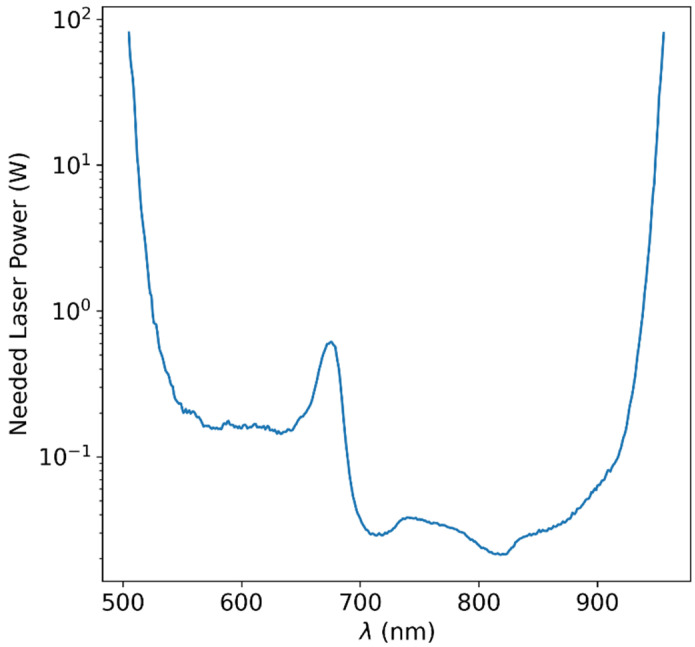
Calculated required LASER power for achieving readable signal at the detector, based on the measured optical attenuation data and the technical specification of common high-end scientific spectroscopic instrumentation. It is clear that at some wavelength ranges, a power of tens of mW will suffice, while at other wavelengths, higher orders of power magnitudes would be required in order to overcome the tissue optical attenuation.

We will use a typical value for a Raman cross-section, $\frac{d\sigma_{raman}}{d\Omega }=10^{-30}\;cm^{2}sr^{-1}$, and since the most abundant component in fruits is usually water, we will use its density as the highest possible value, $\rho_{0}=3.3\times 10^{22}cm^{-3}$. This will be most relevant when measuring systematic changes to the fruit. When trying to sense concentration changes of some specific substance, the final power value may need to be rescaled according to its relative abundance in the fruit. In order to simplify the calculation, we will estimate the light collected as if it was entirely collected from the center of an 8 cm diameter fruit by a 2” lens placed 3 cm from it. By integrating over the differential cross-section and again using the law of sines, we obtain the following:

(10) $$\sigma_{raman}=\int_{0}^{2\pi }\int_{0}^{\bar{\alpha }}\sin\theta \;d\theta d\varphi \;\frac{d\sigma_{raman}}{d\Omega }=\frac{d\sigma_{raman}}{d\Omega }2\pi \left[1-\cos\bar{\alpha }\right]$$

where $\bar{\alpha }=\arctan\frac{r_{lens}}{r_{0}+d_{opt}}$ and $r_{lens}$ is the radius of the lens (we will set it to $=r_{opt}$), $r_{0}$ the radius of the fruit as before and $d_{opt}$ is the distance of the lens from the edge of the fruit. Values of $W_{0}$ as a function of wavelength are presented in Figure 6. We are able to deduce that the optical power needed in the wavelength range that we deduced to be feasible in Table 1 and Table 2 (700–950 nm) is less than 100 mW in the entire range and below 40 mW in most of this range.

Next, we need to discuss the top threshold for the LASER power so it will not damage our samples. For simplicity, let us consider heat conduction in a 1D case. A LASER beam with $r_{opt}$ radius is radiated on one surface of the fruit, and the heat spreads throughout the tissue, forming a temperature gradient that is assumed to be strong enough so that in the steady state, the far side of the fruit is still at room temperature. Let us consider a temperature increase of the irradiated surface of ΔT=50 K, which should not be very harmful at short periods (several seconds) and can also be beneficial as a disinfection treatment [51], and let us also assume that the heat conductivity is close to that of water, the main constituent. The heat conduction equation is as follows:

(11) $$I_{0}=k_{water}\frac{\Delta T}{2r_{0}}$$

where $k_{water}=0.6089\;Wm^{-1}K^{-1}$ is the heat conductivity of water. From this equation, we can deduce that $W_{0}≅0.771\;W$ should be the maximum power of the laser for the above-mentioned parameters ($r_{0}=4\;cm$, $r_{opt}=1^{\prime \prime }$).

**Figure 7:**
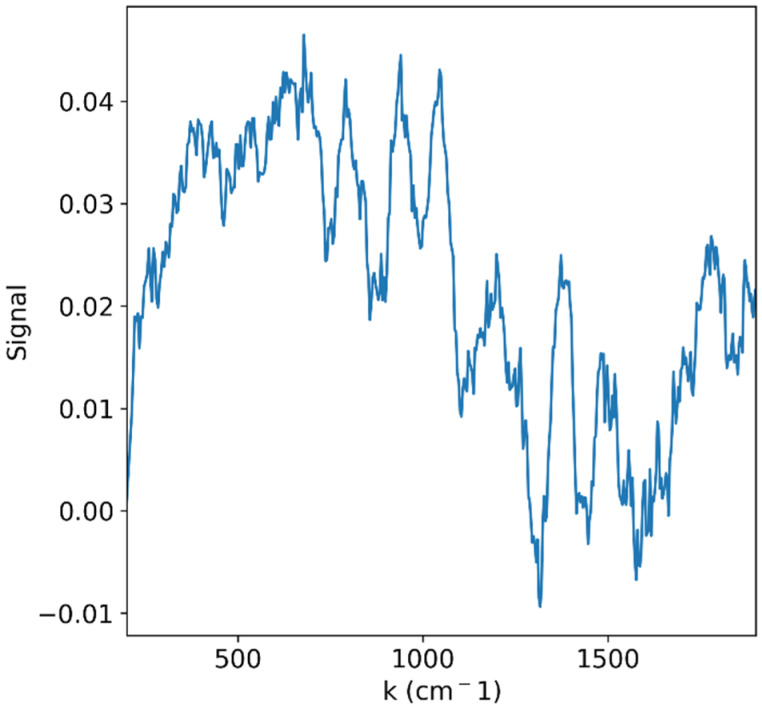
Raman signal of grape slices measured with a 785 nm LASER. The signal is dominated by broad fluorescent features that hinder proper vibrational spectral analysis.

Unfortunately, even if a sufficient Raman signal would reach the detector without damaging the sample, it would still compete with a stronger fluorescence signal. Figure 7 shows an example of such a problem. The Raman spectrum of grape slices was measured with a 785 nm LASER coupled to a Princeton Instruments FERGIE spectrometer (Princeton Instruments, Trenton, NJ, USA) [46]. In spite of improvements to the signal-to-noise ratio due to repeated measurements, the signal is still dominated by a very wide curve and thus is of low quality.

**Figure 8:**
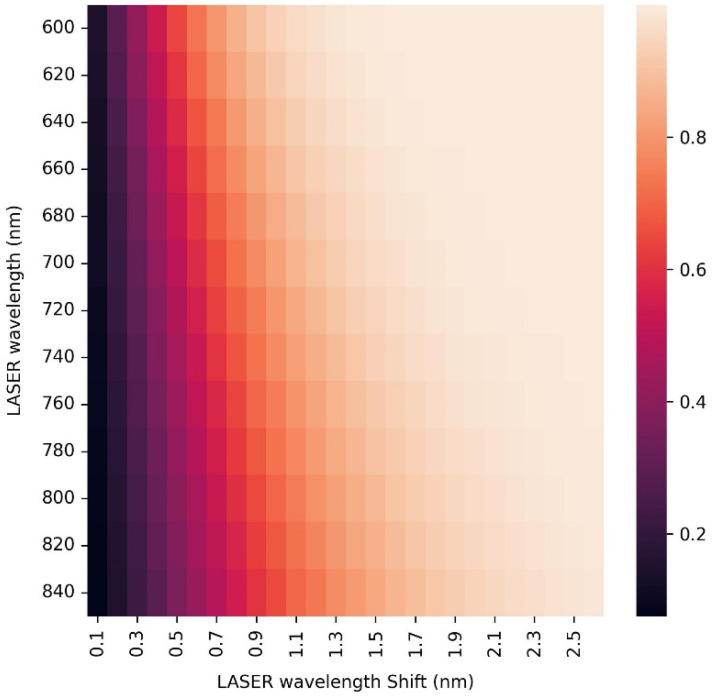
Heat map that describes the dependence of the SERDS peak amplitude on the LASER wavelength and its shift from the second excitation wavelength.

There are several methods of overcoming this problem. Some numerical, such as baseline corrections, can greatly improve results but cannot totally recreate the original Raman signal, and some experimental such as coherent anti-Stokes Raman spectroscopy, which, due to the delicate nature of the biological sample and the pulses high radiation intensity, is not feasible. Another more suitable experimental method is the shifted-excitation Raman difference spectroscopy (SERDS). In this method, the user measures the spectrum twice, each time using one of two closely tuned LASER sources (or one source that can bounce between two wavelengths), and calculates the difference between the two measurements. The fluorescence response is identical in the wavelength axis, and due to the very similar LASER excitation wavelengths, it has nearly equal amplitude and can be eliminated. The Raman response, on the other hand, shifts in the wavelength axis according to the excitation wavelength. If the wavelength difference was chosen correctly, then after the mentioned subtraction, the resulting signal will be in a form similar to a peak derivative that can be numerically integrated in order to obtain the usual peak form. The downsides of this method are the more complex and expensive apparatus consisting of two LASERs instead of one and the broadening of the peaks by the energy difference of the excitation wavelengths. The use of two LASERs can also require the choice of more stable and expensive LASERs or a custom source [52,53,54,55]. Using again the spectral width of $W_{k}=30\;cm^{-1}$ that was measured with FTIR, and the desired spectral resolution of $\Delta k=3\;cm^{-1}$ that was deduced from it, we can calculate the ideal excitation wavelength difference for any choice of LASER and spectral region in order to reduce the peak broadening as much as possible while increasing the final subtracted peak intensity. Figure 8 shows the dependence of the resulting integrated peak amplitude (centered around $1100\;cm^{-1}$ and normalized) for different excitation LASER wavelengths and LASER wavelength shifts. It is clear that when choosing the smallest LASER wavelength shifts for the highest spectral resolution, the signal amplitude diminishes. It is also clear that it will not be beneficial to go beyond certain LASER shifts for each excitation wavelength since the peak’s amplitude will not increase, but its width will. While in the wavelength domain, this width will be the LASER wavelength shift ($\Delta \lambda_{laser}$), in the wave number domain the width will be as follows (using Equation (2)):

(12) $$\Delta k=\frac{10^{7}\Delta \lambda_{laser}}{{\left(\lambda_{laser}\right)}^{2}}$$

Similar investigations produced no dependency of the signal amplitude on the energy of the mode. For example, let us choose the excitation wavelength of ~695 nm since at this wavelength, it is very likely to find a commercial LASER diode with very high power (several hundreds of mW) [56,57,58,59], and the above parameters of a spectral peak in the middle of the fingerprint region. At this excitation wavelength, in order to obtain the maximum peak amplitude and minimal width, the LASER shift should be 1.7 nm, meaning two excitations at 695 and 696.7 nm (we can tune both diodes to longer wavelengths in order to experience less optical attenuation) [60,61]. This will result in additional spectral broadening of $\sim 35\;cm^{-1}$. We should mention, though, that one can compromise on a 90% peak amplitude at a LASER shift of 1 nm so that the broadening will be only $\sim 20\;cm^{-1}$. We should also mention that this wavelength difference is much higher than the linewidth of the LASER itself, which at the most has a linewidth <0.1 nm for Raman designated LASERs.

## 3. Discussion

There are several options for realizing a double wavelength LASER source. If there is no need for multiple completely different excitation wavelengths for different specimens, one can purchase and use LASER diodes with specific wavelengths, which today are the most commercially common and cheap sources. Since the desired SERDS wavelength difference is found here to be small (~1 nm), it is possible to use one LASER diode device and tune it by changing its temperature for each measurement [62]. However, this will make the measurement very slow since one will have to wait for temperature stabilization and can make the measurement unfeasible when dealing with sensitive live specimens. To overcome this problem, it is possible to use two identical diode sources and adjust their temperature via their controller in order to shift one or both wavelengths to match the desired wavelengths. One will, however, need to use spectral notch filters and neutral density filters in order to have equal intensity narrow linewidth sources since these tunable diodes will not produce light that is spectrally narrow enough for Raman spectroscopy. A more practical approach may be to acquire two similar wavelengths, non-tunable LASERs. Often, these LASERs’ wavelengths differ from one another in a certain range, and it is possible to find a pair with the desired wavelength difference. Combining the LASER beams can be achieved by controlling their polarization and using a polarizing beam splitter (PBS) [63], or if using cheaper, non-polarized sources, by using a spliced or bifurcated fiber combiner. Highly tunable sources were also used [64], but their price tag is much higher, and perhaps it is more worthwhile to operate multiple sources and combine them in a more complex optical setup. Finally, there are also commercial SERDS sources for specific wavelengths that provide easier operation and robustness [65,66].

**Figure 9:**
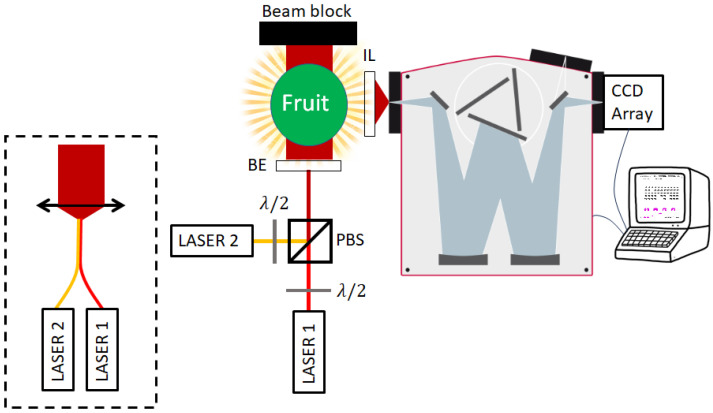
Schematics for the proposed transmission Raman measurement system. Two closely tuned LASERs are combined to the same optical path by controlling their polarization relative to the PBS. The resulting beam is expanded and passed through the sample. The sample is imaged onto the monochromator’s slit, and the light is then dispersed on the pixels of the CCD. Another option for producing the combined optical beam is displayed inside the dashed rectangle—two fiber-outputted LASERs are combined with a spliced or bifurcated fiber combiner, and a plano-convex lens is used to collimate the beam to the desired expanded width. See Section 3 for the full details.

Taking into consideration the above points and calculations, we can propose a design for the measurement system that should fit most above discussed applications. This design is shown in Figure 9. The polarizations of two LASER diodes’ collimated beams are adjusted to be perpendicular to one another and combined with PBS. The resulting beam is optically expanded almost to the width of the fruit and interacts with it. The fruit and the resulting light field are then imaged onto the monochromator slit using an imaging lens (IL), after which the light is angularly separated according to the different wavelengths and reflected onto separate pixels of a CCD array detector, which are read into a connected computer that can also control the monochromator. Another option for producing the combined optical beam is displayed inside the dashed rectangle—two fiber-outputted LASERs are combined with a spliced or bifurcated fiber combiner, and a plano-convex lens is used to collimate the beam to the desired expanded width.

The design and set of considerations brought here can be used not only on apples and other water-based fruits with slight wavelength shifts due to different attenuation curves or skin color but on other biological samples as well. The governing principles are the location of the optimal spectral window, the irradiation with the maximum allowed intensity and above the minimal detection threshold, and the elimination of the fluorescence signal contributions. These other biological samples can ideally be any object with enough volume to justify the requirement of an optical window, but besides being other fruit or vegetables such as potato, mango, pear, plum, etc., they can also be meat, fish, and small cheese wheels. Each of these options has its own optical window, and here lies the challenge of having and locating a window that will be wide enough to pass through all of the required data and transparent enough that it would be possible to find the LASER with the suitable wavelength with enough power to pass through the material. It is important, though, to directly address the case that is presented in Figure 1 of the bell pepper, regarding the math in this paper. In the bell pepper case, if we consider the cuticle and epidermis layers as the “peel” layer and only take into consideration the Raman signal originating from the outer layer of the placenta as it will be the strongest contribution to the total Raman signal, the output radiation power will become the following:

(13) $$P^{\prime }=I_{0}\rho_{0}\sigma_{raman}\pi r_{opt}^{2}Q_{peel}^{2}$$

The thermal calculation becomes a problem that is solved across the shell of the bell pepper since the inside is mostly insulating air. Therefore, by taking the total optical power and transmitting it through the layer adjacent to the laser spot across the bell pepper’s shell, we obtain the following:

(14) $$I_{0}=\frac{d_{peel}}{r_{opt}}k_{water}\frac{\Delta T}{\pi r_{0}}$$

Since $\frac{d_{peel}}{r_{opt}}≅0.2$ and $\frac{2}{\pi }≅0.64$, we can come to the conclusion that the maximum allowed power will also be reduced by a factor of ~8.

Another key aspect of making this technology work for the user is the ability to decipher the data. Since we will be measuring raw biological tissues without any physical preprocessing and phase separations, each acquired spectrum will contain many components from different chemical substances, each with its own peak locations and intensities, and each substance has its own abundance. The ideal goal here is to be able to tell the abundance of each component in the tissue, which is very challenging and has not been achieved yet because of the complexity of the data. One option to satisfy the user is to provide him with only the data that he is interested in, for example, sugar content. This has been performed many times with changing degrees of success in the near IR/SWIR spectral regimes by implementing principal component analysis schemes such as Partial Least Squares regression [67,68]. However, due to the very wide spectral features at this range, one will mostly measure the overtones and combination bands of the water component, and any correlation with other carbon-based components (which are plentiful) will probably be determined by parameters like the solubility of these substances in water [47]. In the scheme described in this paper, the measured data complexity is expected to be much higher than these SWIR attempts due to the higher spectral resolution compared with the common InGaAs-based detector used for SWIR, and since the spectral peak is expected to be from much purer and not harmonics or combined modes, it is possible to implement much more advanced and modern analysis schemes. The most promising of these is probably using neural networks. Since this method requires many training data, the idea is to create a vast synthetic spectral database while controlling its spectral data complexity by controlling the line shapes of the spectral peaks, the intensity of a random background curve, the number of known materials, the complexity of the known materials spectra, the number of phases in the complex spectra, and the amount of noise. The data from the synthetic database would then be hot encoded and fed into a neural network for training and testing.

This will allow the testing of different layer topologies in order to maximize accuracy. The layer structure should be composed of several convolution layers stacked with a variable number of filters and kernel sizes, each followed by a pooling layer. The data will then be flattened and reduced through several fully connected layers, where the last layer corresponds to the number of known materials with a non-exclusive classification activation function. After achieving a sufficient level of accuracy, it will be possible to use a small number of measured spectra whose specimens were measured separately with other analytical instrumentation in order to identify their composing materials and quantities in order to train the last layer of the network and adjust it better to real-world data. This analysis method will be developed and tested in future work. More simple applications for these combined measurement and analysis techniques are of the binary nature such as the identification of core rot and off-flavor that were mentioned in the introduction. Tackling these issues would require much less measured data, thus enabling the elimination of damaged produce more easily.

## 4. Conclusions

In this paper, we have presented optical attenuation measurements of apples and used them in order to design an optical Raman system for transmission measurements on biological samples as large as several cm, such as apples. This was combined with numerical calculations in order to assess the correct optical and physical parameters that will help the success of these types of measurements. In addition, we have discussed other optical design and data analysis aspects that will allow most users to successfully use this method. Further work that can be performed in the future in order to advance this technique was also mentioned. The successful implementation and adoption of this method will allow vast advancements in chemical analysis and properties or problem identification in bulk samples, a field that has not been properly handled as of today, such as internal rot in apples or bell peppers, as presented and discussed here in previous sections using semi-empirical methods.

## Figures and Tables

**Table 1:** Several excitation wavelengths spanning the possible range of 600–825 nm. At each excitation wavelength, the highest wavelength that is needed to be measured is listed in 2 scenarios depending on the span of spectroscopic data needed (1600 cm^−1^ or 3750 cm^−1^), along with the required wavelength dispersion and number of pixels.

		Excitation Wavelength [nm]	Wavelength Range [nm]	Dispersion [nm/pixel]	Number of Pixels
Highest Wavenumber	$3750\;cm^{-1}$	600	774	0.108	1613
		620	808	0.115	1629
		640	842	0.123	1645
		660	877	0.131	1661
		680	913	0.139	1678
		700	949	0.147	1695
	$1600\;cm^{-1}$	725	820	0.158	603
		750	852	0.169	606
		775	885	0.180	609
		800	917	0.192	612
		825	950	0.204	615

**Table 2:** Several spectrometer parameters options for achieving the spectroscopic requirement in Table 1. Each option lists the focal length (f), grating density (ρ), and grating blaze, along with the eventual spectral resolution (Δλ) expected in a spectral region between $\lambda_{i}$ and $\lambda_{f}$, and the minimal expected efficiency at that range (taken from [48]).

f (mm)	ρ (g/mm)	$\lambda_{i}\;(nm)$	$\lambda_{f}\;(nm)$	Δλ (nm)	Blaze	Efficiency
300	300	700	985	0.139	1 μm	>60%
300	600	800	933	0.065	1 μm	>70%
500	300	772	950	0.086	1 μm	>75%
150	600	800	1027	0.111	1 μm	>70%

## Data Availability

The data supporting the conclusions of this work can be obtained at the Mendeley Data server [46].
